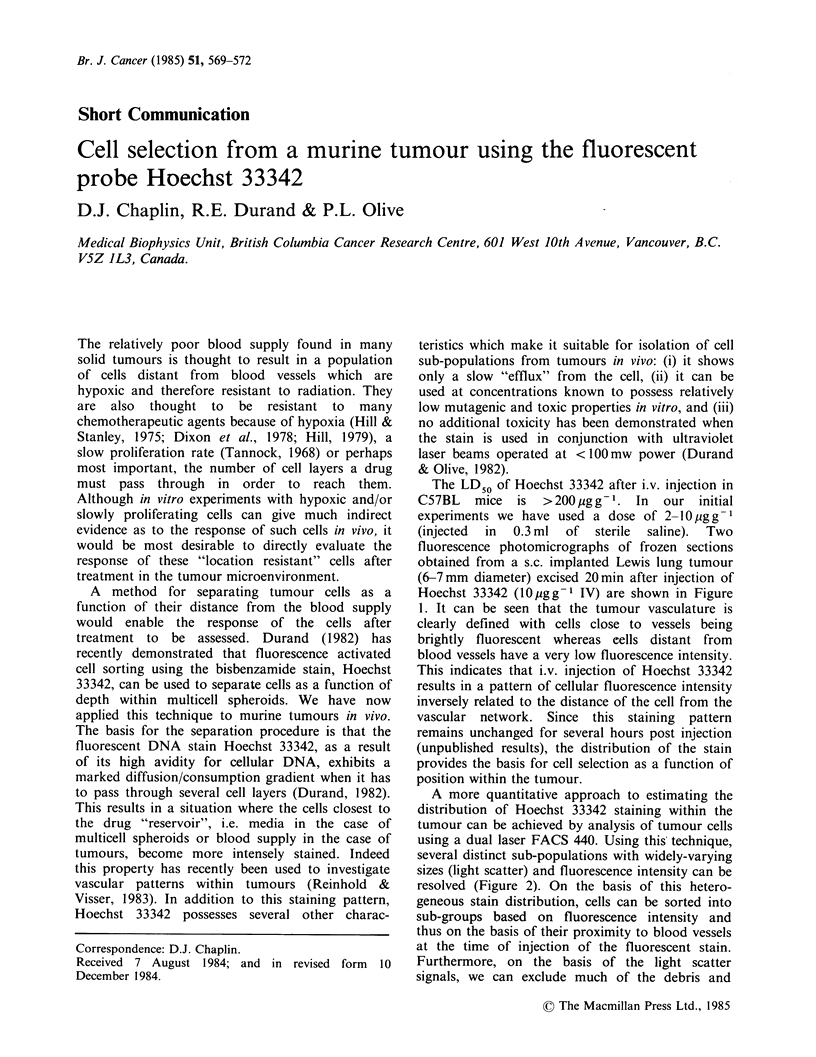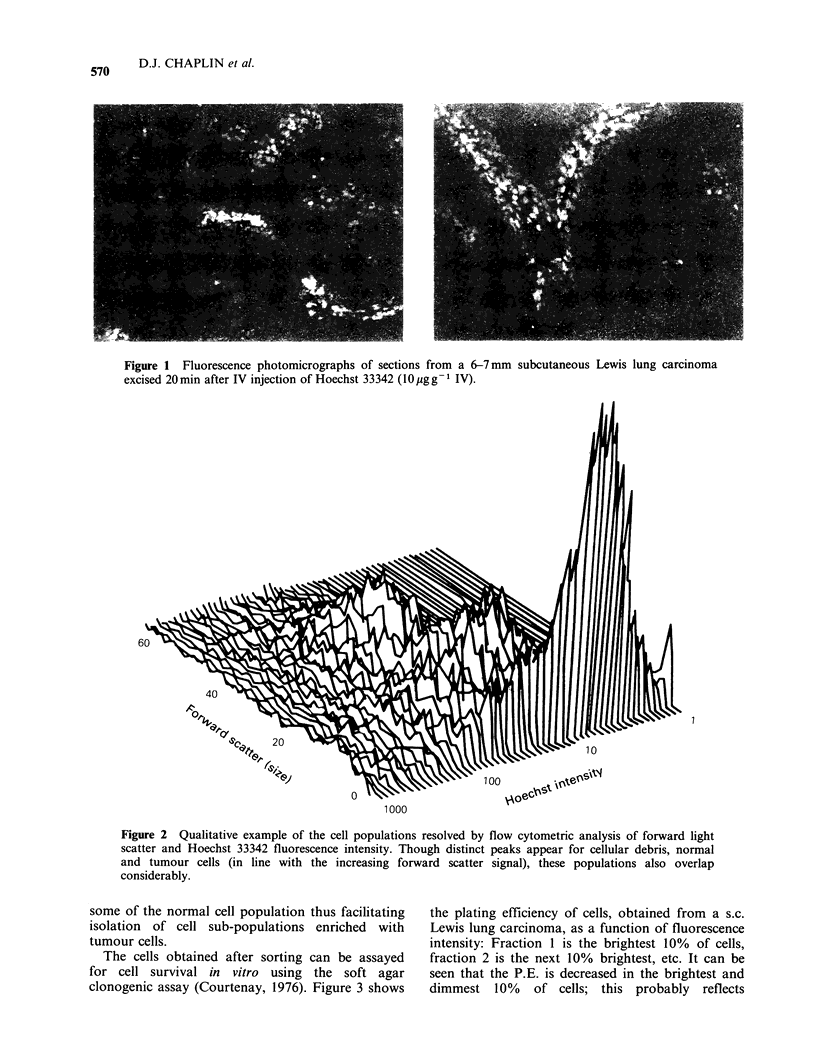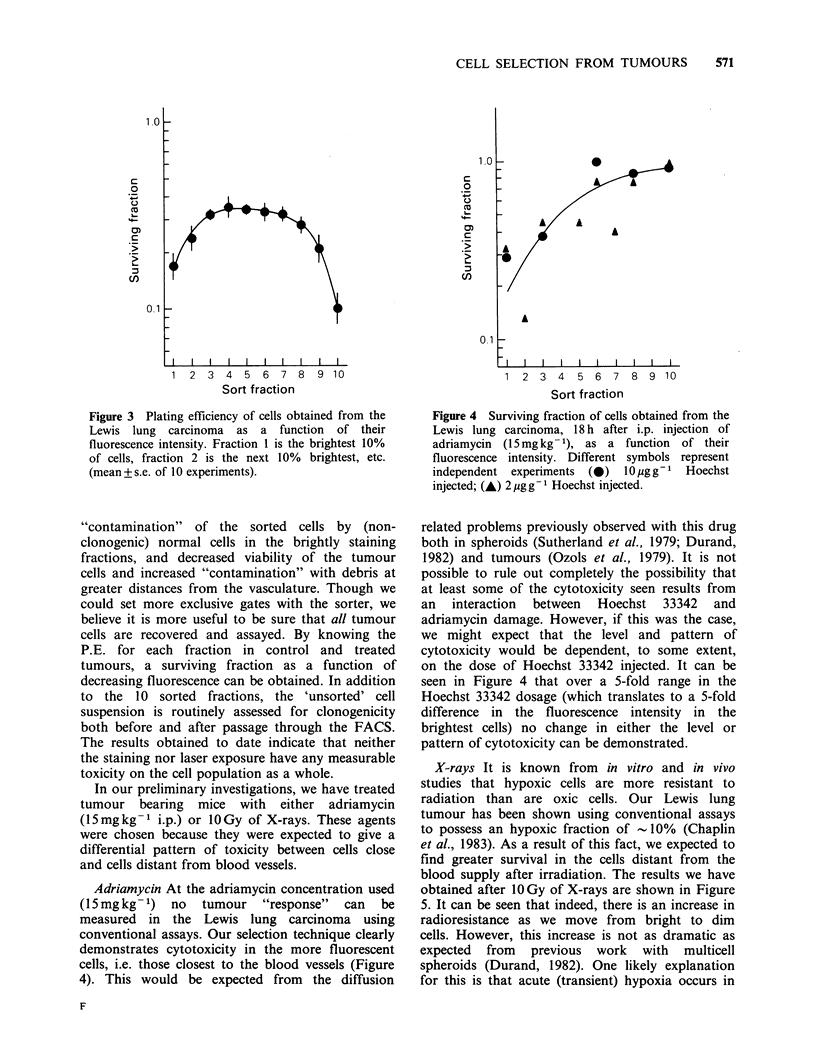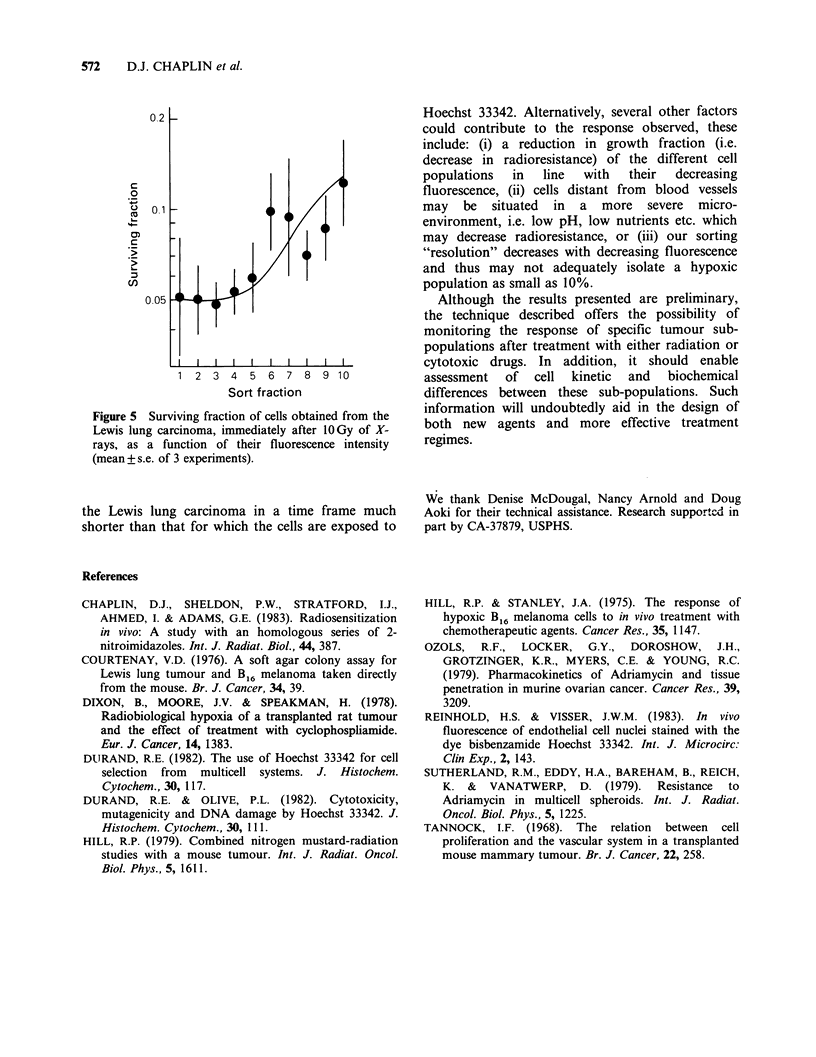# Cell selection from a murine tumour using the fluorescent probe Hoechst 33342.

**DOI:** 10.1038/bjc.1985.79

**Published:** 1985-04

**Authors:** D. J. Chaplin, R. E. Durand, P. L. Olive

## Abstract

**Images:**


*Br. J. Cancer* (1985) **51**, 569–572

**Short Communication**

### Cell selection from a murine tumour using the fluorescent probe Hoechst 33342

D.J. Chaplin, R.E. Durand & P.L. Olive

*Medical Biophysics Unit, British Columbia Cancer Research Centre, 601 West 10th Avenue, Vancouver, B.C. V5Z 1L3, Canada.*

The relatively poor blood supply found in many solid tumours is thought to result in a population of cells distant from blood vessels which are hypoxic and therefore resistant to radiation. They are also thought to be resistant to many chemotherapeutic agents because of hypoxia (Hill & Stanley, 1975; Dixon *et al.*, 1978; Hill, 1979), a slow proliferation rate (Tannock, 1968) or perhaps most important, the number of cell layers a drug must pass through in order to reach them. Although *in vitro* experiments with hypoxic and/or slowly proliferating cells can give much indirect evidence as to the response of such cells *in vivo*, it would be most desirable to directly evaluate the response of these "location resistant" cells after treatment in the tumour microenvironment.

A method for separating tumour cells as a function of their distance from the blood supply would enable the response of the cells after treatment to be assessed. Durand (1982) has recently demonstrated that fluorescence activated cell sorting using the bisbenzamide stain, Hoechst 33342, can be used to separate cells as a function of depth within multicell spheroids. We have now applied this technique to murine tumours *in vivo*. The basis for the separation procedure is that the fluorescent DNA stain Hoechst 33342, as a result of its high avidity for cellular DNA, exhibits a marked diffusion/consumption gradient when it has to pass through several cell layers (Durand, 1982). This results in a situation where the cells closest to the drug "reservoir", i.e. media in the case of multicell spheroids or blood supply in the case of tumours, become more intensely stained. Indeed this property has recently been used to investigate vascular patterns within tumours (Reinhold & Visser, 1983). In addition to this staining pattern, Hoechst 33342 possesses several other characteristics which make it suitable for isolation of cell sub-populations from tumours *in vivo*: (i) it shows only a slow "efflux" from the cell, (ii) it can be used at concentrations known to possess relatively low mutagenic and toxic properties *in vitro*, and (iii) no additional toxicity has been demonstrated when the stain is used in conjunction with ultraviolet laser beams operated at $<100$ mw power (Durand & Olive, 1982).

The $LD_{50}$ of Hoechst 33342 after i.v. injection in C57BL mice is $>200\,\mu g\,g^{-1}$. In our initial experiments we have used a dose of 2–10 $\mu g\,g^{-1}$ (injected in 0.3 ml of sterile saline). Two fluorescence photomicrographs of frozen sections obtained from a s.c. implanted Lewis lung tumour (6–7 mm diameter) excised 20 min after injection of Hoechst 33342 (10 $\mu g\,g^{-1}$ IV) are shown in Figure 1. It can be seen that the tumour vasculature is clearly defined with cells close to vessels being brightly fluorescent whereas cells distant from blood vessels have a very low fluorescence intensity. This indicates that i.v. injection of Hoechst 33342 results in a pattern of cellular fluorescence intensity inversely related to the distance of the cell from the vascular network. Since this staining pattern remains unchanged for several hours post injection (unpublished results), the distribution of the stain provides the basis for cell selection as a function of position within the tumour.

A more quantitative approach to estimating the distribution of Hoechst 33342 staining within the tumour can be achieved by analysis of tumour cells using a dual laser FACS 440. Using this technique, several distinct sub-populations with widely-varying sizes (light scatter) and fluorescence intensity can be resolved (Figure 2). On the basis of this heterogeneous stain distribution, cells can be sorted into sub-groups based on fluorescence intensity and thus on the basis of their proximity to blood vessels at the time of injection of the fluorescent stain. Furthermore, on the basis of the light scatter signals, we can exclude much of the debris and

Correspondence: D.J. Chaplin.
Received 7 August 1984; and in revised form 10 December 1984.

© The Macmillan Press Ltd., 1985

**Figure 1** Fluorescence photomicrographs of sections from a 6–7 mm subcutaneous Lewis lung carcinoma excised 20 min after IV injection of Hoechst 33342 (10 $\mu g\, g^{-1}$ IV).

**Figure 2** Qualitative example of the cell populations resolved by flow cytometric analysis of forward light scatter and Hoechst 33342 fluorescence intensity. Though distinct peaks appear for cellular debris, normal and tumour cells (in line with the increasing forward scatter signal), these populations also overlap considerably.

some of the normal cell population thus facilitating isolation of cell sub-populations enriched with tumour cells.

The cells obtained after sorting can be assayed for cell survival *in vitro* using the soft agar clonogenic assay (Courtenay, 1976). Figure 3 shows the plating efficiency of cells, obtained from a s.c. Lewis lung carcinoma, as a function of fluorescence intensity: Fraction 1 is the brightest 10% of cells, fraction 2 is the next 10% brightest, etc. It can be seen that the P.E. is decreased in the brightest and dimmest 10% of cells; this probably reflects

**Figure 3** Plating efficiency of cells obtained from the Lewis lung carcinoma as a function of their fluorescence intensity. Fraction 1 is the brightest 10% of cells, fraction 2 is the next 10% brightest, etc. (mean ± s.e. of 10 experiments).

**Figure 4** Surviving fraction of cells obtained from the Lewis lung carcinoma, 18 h after i.p. injection of adriamycin ($15\,mg\,kg^{-1}$), as a function of their fluorescence intensity. Different symbols represent independent experiments (●) $10\,\mu g\,g^{-1}$ Hoechst injected; (▲) $2\,\mu g\,g^{-1}$ Hoechst injected.

"contamination" of the sorted cells by (non-clonogenic) normal cells in the brightly staining fractions, and decreased viability of the tumour cells and increased "contamination" with debris at greater distances from the vasculature. Though we could set more exclusive gates with the sorter, we believe it is more useful to be sure that *all* tumour cells are recovered and assayed. By knowing the P.E. for each fraction in control and treated tumours, a surviving fraction as a function of decreasing fluorescence can be obtained. In addition to the 10 sorted fractions, the 'unsorted' cell suspension is routinely assessed for clonogenicity both before and after passage through the FACS. The results obtained to date indicate that neither the staining nor laser exposure have any measurable toxicity on the cell population as a whole.

In our preliminary investigations, we have treated tumour bearing mice with either adriamycin ($15\,mg\,kg^{-1}$ i.p.) or 10 Gy of X-rays. These agents were chosen because they were expected to give a differential pattern of toxicity between cells close and cells distant from blood vessels.

*Adriamycin* At the adriamycin concentration used ($15\,mg\,kg^{-1}$) no tumour "response" can be measured in the Lewis lung carcinoma using conventional assays. Our selection technique clearly demonstrates cytotoxicity in the more fluorescent cells, i.e. those closest to the blood vessels (Figure 4). This would be expected from the diffusion related problems previously observed with this drug both in spheroids (Sutherland *et al.*, 1979; Durand, 1982) and tumours (Ozols *et al.*, 1979). It is not possible to rule out completely the possibility that at least some of the cytotoxicity seen results from an interaction between Hoechst 33342 and adriamycin damage. However, if this was the case, we might expect that the level and pattern of cytotoxicity would be dependent, to some extent, on the dose of Hoechst 33342 injected. It can be seen in Figure 4 that over a 5-fold range in the Hoechst 33342 dosage (which translates to a 5-fold difference in the fluorescence intensity in the brightest cells) no change in either the level or pattern of cytotoxicity can be demonstrated.

*X-rays* It is known from *in vitro* and *in vivo* studies that hypoxic cells are more resistant to radiation than are oxic cells. Our Lewis lung tumour has been shown using conventional assays to possess an hypoxic fraction of ~10% (Chaplin *et al.*, 1983). As a result of this fact, we expected to find greater survival in the cells distant from the blood supply after irradiation. The results we have obtained after 10 Gy of X-rays are shown in Figure 5. It can be seen that indeed, there is an increase in radioresistance as we move from bright to dim cells. However, this increase is not as dramatic as expected from previous work with multicell spheroids (Durand, 1982). One likely explanation for this is that acute (transient) hypoxia occurs in

**Figure 5** Surviving fraction of cells obtained from the Lewis lung carcinoma, immediately after 10 Gy of *X*-rays, as a function of their fluorescence intensity (mean ± s.e. of 3 experiments).

the Lewis lung carcinoma in a time frame much shorter than that for which the cells are exposed to Hoechst 33342. Alternatively, several other factors could contribute to the response observed, these include: (i) a reduction in growth fraction (i.e. decrease in radioresistance) of the different cell populations in line with their decreasing fluorescence, (ii) cells distant from blood vessels may be situated in a more severe micro-environment, i.e. low pH, low nutrients etc. which may decrease radioresistance, or (iii) our sorting "resolution" decreases with decreasing fluorescence and thus may not adequately isolate a hypoxic population as small as 10%.

Although the results presented are preliminary, the technique described offers the possibility of monitoring the response of specific tumour sub-populations after treatment with either radiation or cytotoxic drugs. In addition, it should enable assessment of cell kinetic and biochemical differences between these sub-populations. Such information will undoubtedly aid in the design of both new agents and more effective treatment regimes.

We thank Denise McDougal, Nancy Arnold and Doug Aoki for their technical assistance. Research supported in part by CA-37879, USPHS.

### References

CHAPLIN, D.J., SHELDON, P.W., STRATFORD, I.J., AHMED, I. & ADAMS, G.E. (1983). Radiosensitization *in vivo*: A study with an homologous series of 2-nitroimidazoles. *Int. J. Radiat. Biol.*, **44**, 387.

COURTENAY, V.D. (1976). A soft agar colony assay for Lewis lung tumour and $B_{16}$ melanoma taken directly from the mouse. *Br. J. Cancer*, **34**, 39.

DIXON, B., MOORE, J.V. & SPEAKMAN, H. (1978). Radiobiological hypoxia of a transplanted rat tumour and the effect of treatment with cyclophospliamide. *Eur. J. Cancer*, **14**, 1383.

DURAND, R.E. (1982). The use of Hoechst 33342 for cell selection from multicell systems. *J. Histochem. Cytochem.*, **30**, 117.

DURAND, R.E. & OLIVE, P.L. (1982). Cytotoxicity, mutagenicity and DNA damage by Hoechst 33342. *J. Histochem. Cytochem.*, **30**, 111.

HILL, R.P. (1979). Combined nitrogen mustard-radiation studies with a mouse tumour. *Int. J. Radiat. Oncol. Biol. Phys.*, **5**, 1611.

HILL, R.P. & STANLEY, J.A. (1975). The response of hypoxic $B_{16}$ melanoma cells to *in vivo* treatment with chemotherapeutic agents. *Cancer Res.*, **35**, 1147.

OZOLS, R.F., LOCKER, G.Y., DOROSHOW, J.H., GROTZINGER, K.R., MYERS, C.E. & YOUNG, R.C. (1979). Pharmacokinetics of Adriamycin and tissue penetration in murine ovarian cancer. *Cancer Res.*, **39**, 3209.

REINHOLD, H.S. & VISSER, J.W.M. (1983). *In vivo* fluorescence of endothelial cell nuclei stained with the dye bisbenzamide Hoechst 33342. *Int. J. Microcirc: Clin Exp.*, **2**, 143.

SUTHERLAND, R.M., EDDY, H.A., BAREHAM, B., REICH, K. & VANATWERP, D. (1979). Resistance to Adriamycin in multicell spheroids. *Int. J. Radiat. Oncol. Biol. Phys.*, **5**, 1225.

TANNOCK, I.F. (1968). The relation between cell proliferation and the vascular system in a transplanted mouse mammary tumour. *Br. J. Cancer*, **22**, 258.